# Do Older Women of Reproductive Age Have Better Diet Quality than Younger Women of Reproductive Age?

**DOI:** 10.3390/nu13113830

**Published:** 2021-10-27

**Authors:** Nahal Habibi, Katherine M. Livingstone, Suzanne Edwards, Jessica A. Grieger

**Affiliations:** 1 Robinson Research Institute, University of Adelaide, Adelaide, SA 5005, Australia; nahal.habibi@adelaide.edu.au; 2 Adelaide Medical School, University of Adelaide, Adelaide, SA 5005, Australia; 3 Institute for Physical Activity and Nutrition, School of Exercise and Nutrition Sciences, Deakin University, Geelong, VIC 3220, Australia; k.livingstone@deakin.edu.au; 4 Adelaide Health Technology Assessment (AHTA), University of Adelaide, Adelaide, SA 5005, Australia; suzanne.edwards@adelaide.edu.au

**Keywords:** Australia, dietary guidelines, dietary guideline index, dietary intake, nutrients, reproductive age, women, nutrition survey

## Abstract

There is increasing recognition of the importance of nutrition for reproductive health, but little is known regarding the diet quality of younger vs. older reproductive aged women, and how their intakes relate to dietary recommendations. The purpose of the study was to examine the diets of younger (19–35 years old) compared to older (35–50 years old) reproductive aged women, and how they align with dietary recommendations. Women aged 19–50 years from the 2011–13 Australian National Nutrition and Physical Activity Survey were included (*n* = 2323). Dietary intakes were assessed by a single 24-h dietary recall and were compared to (i) Australian Dietary Guidelines; (ii) Acceptable Macronutrient Distribution for protein, carbohydrates, and fat; and (iii) Dietary Guideline Index (DGI). Regression analyses comparing younger and older women against recommendations were undertaken, with confounders determined a priori. There was no difference between older and younger women in meeting food group recommendations, with 26% of all women meeting recommendations for fruit, and meat and alternatives, and <20% meeting recommendations for vegetables and alternatives, grains, and dairy. Although there was no difference between older and younger women in total DGI score (mean (SE) 75.6 (1.7) vs. 74.5 (2.5), *p* > 0.05), older women had higher component scores in limiting saturated fat, consuming low-fat milk, and limiting adding salt during cooking. Continued health promotion for women of reproductive age should be a key priority to improve their own health and that of future generations.

## 1. Introduction

Over the last several decades, worldwide fertility rates have declined across all age groups, with largest decreases occurring in women younger than 35 years, whereas those aged 35 years and over effectively plateauing since 1995 [1]. Childbearing over 35 years of age is increasingly common in Australia [2], with around 20% of births in women aged 35 years and over [3].

There is increasing recognition of the importance of nutrition for reproductive health [4]. Observational studies have consistently shown associations between poorer food choices or unhealthier dietary patterns and higher risk for infertility [5,6,7], gestational diabetes (reviewed in [8,9]) and preterm birth [10], but also contributing to poorer outcomes for the offspring, including increased risk for low birth weight [11], child allergy [12], and child adiposity [13].

Reproductive life stages include the preconception period, pregnancy and postpartum. Across any of the specific stages, studies have demonstrated inadequate dietary quality [14,15,16,17]. However, little is known about food intake during childbearing years and whether this differs between younger and older age groups. Data from the Australian Longitudinal Study on Women’s Health (*n* = 18,226) found that the majority of women (aged 31–36 years or 50–55 years), tended to consume intakes below the Australian recommended daily servings for all food groups, except for fruit intake, among pregnant women aged 31–36 years [18]. Women aged 25 to 30 years who had given birth in the last 12 months also reported to have greater median daily servings of breads and cereals, vegetables, dairy, meat and extras (i.e., foods outside of the core/basic five food groups) compared to women not trying to, or women who were recently pregnant [19]. Data from NHANES women aged 15–65 years (*n* = 6894), found that irrespective of age, more than half of the women were at risk of nutrient inadequacy, with insufficient intakes from food for vitamin D, vitamin E, magnesium, vitamin A, calcium, and vitamin C [20]. While compliance with dietary guidelines provides insight into dietary habits and population intakes, assessing diet quality within populations provides a holistic assessment of food intake and nutrient adequacy. Few studies however have reported on this in women of reproductive age. A small study in Australian women reported no difference in total diet quality between urban and rural women of reproductive age, aged between 18–50 years [21].

Reproductive aged women are in a critical life stage and have distinct and specific nutritional needs. They play diverse roles including planning or transitioning during pregnancies [22], being a role model to their children [4], and they are more likely to prepare meals for their family [5]. Women of reproductive age contribute to the highest rise in obesity prevalence [23], and also have increasing prevalence of other chronic disease–related risk factors such as diabetes, high cholesterol, and asthma [24]. Yet, we have little understanding regarding the diet quality of these women, nor how their intakes relate to dietary recommendations. We hypothesise that older women will be more likely to meet dietary recommendations and have better diet quality than younger women. The aim of this study is to examine how the diets of younger and older reproductive aged women participating in the 2011–13 Australian Health Survey compare with current food group recommendations and with the Dietary Guideline Index (DGI), as a means to understand overall diet quality. A secondary aim is to explore whether the younger and older age women who have children, have different diet quality compared to women without children.

## 2. Materials and Methods

### 2.1. Data and Study Population

Data was used from a sub-set of women participating in the 2011–13 Australian Health Survey: the Australian National Nutrition and Physical Activity Survey (NNPAS) [25]. In the NNPAS, a total of 14,363 private dwellings were selected in the sample (reduced to an actual sample of 12,366 dwellings after sample loss in the field stage), in which 77.0% were fully or adequately responding households to the first interview (*n* = 9519). Inclusion criteria for the current study was females aged 19 to 50 years and currently menstruating (*n* = 2323). Women were excluded if they were pregnant or breastfeeding as nutritional requirements are generally higher for these women (*n* = 228) [26], or if they were current or post-menopausal (*n* = 1993). Women were split into two groups: younger women aged 19 to 35 years, and women of advanced age, >35–50 years. The Census and Statistics Act, 1905 provided the Australian Bureau of Statistics with the authority to conduct NNPAS, with all respondents providing written informed consent.

Sociodemographic variables including age, country of birth (Australia; main English-speaking countries [Canada, Ireland, New Zealand, South Africa, UK, USA]; other), household type (person living alone; couple only; couple family with children; one parent family with children; unrelated persons aged 15+ only, all other households), education, and anthropometric data (height, weight) were collected by trained interviewers. Socioeconomic status was based on the Index of Relative Socio-Economic Disadvantage (IRSD). The IRSD ranks Australian areas according to relative socioeconomic disadvantage, obtained from four indices of disadvantage including low income, low educational achievement, high unemployment, and jobs in relatively unskilled occupations [25]. Smoking status was defined as daily, weekly or less than weekly current smoker, ex-smoker or never smoked. Physical activity was reported as whether individuals met the minimum recommendation of moderate intensity of physical activity for 150 min during the last week [27]. Supplement intake, and food and beverage intake data were collected using a 24-h recall, as described below.

### 2.2. Dietary Intake

In the survey, 2 × 24-h dietary recalls were administered by trained and experienced interviewers using the Automated Multiple-Pass Method (AMPM), to collect dietary information for food, beverages, and supplements. The AMPM method is an automated questionnaire to help respondents maximise responses regarding their prior food intake [28]. A Food Model Booklet was used to assist respondents to select the most appropriate amount consumed for each food and beverage. For the current analysis, only dietary data from the first day of collection was included since a single day’s intake is sufficient to estimate population mean intake [29], and because Friday and Saturday intakes were under-represented due to the lower number of recalls performed on Saturdays and Sundays.

### 2.3. Australian Dietary Recommendations

The Australian Dietary Guidelines recommend food and beverage choices from the five core food groups and to limit discretionary choices [30]. Dietary intake of core food groups including vegetables and legumes/beans, fruits, grains, meat and alternatives (meat and poultry, fish, eggs, tofu, nuts and seeds and legumes/beans) and dairy in servings/day, and discretionary nutrients such as free sugars (% daily energy intake), sodium (mg/day), saturated fatty acids (SFA) (% daily energy intake), and alcohol (g/day) were obtained from the 24-hr recall and examined against the Australian Guide to Healthy Eating (AGHE) [30]. The AGHE defines types and amounts of foods that adult women should consume in order to meet dietary intakes. Macronutrient recommendations were based on the Acceptable Macronutrient Distribution (AMDR) for protein, carbohydrates, and fat [31]. The AMDR describes the acceptable percentage of energy from protein, carbohydrates, and fat as 15–25%, 45–65%, and 20–35% of total daily energy, respectively.

### 2.4. Dietary Guidelines Index

The DGI is a food-based score designed to reflect the diet quality of subjects according to compliance with the 2013 ADG for Australian adults [30]. The dietary intakes gathered from the 24-h recall and brief questionnaire were scored based on recommended dietary components (food variety, fruit, vegetables, cereals, meat and alternatives, dairy and alternatives, and fluid intake) and discretionary nutrients (SFA, unsaturated fat, added salt, extra sugar, and alcohol). The DGI used in the present study was based on the DGI-2013 [32], and adapted from a food frequency questionnaires, for use in the present 24-h recall [33]. The score of each item was calculated out of 10, such that a score of zero indicated that the guideline was not met. Where there was age- or sex-specific dietary recommendations provided by the ADG, cut-offs were used to acquire the maximum score for each component. For recommended dietary components, scores were calculated proportionally to the maximum scoring criteria. Scoring of discretionary foods, saturated and unsaturated fat, salt, sugar, and alcohol was either 0 or 10. The DGI scores ranged from 0 to 130 with a higher score indicating better diet quality.

### 2.5. Statistical Analyses

Throughout the analysis of this study (for both descriptive and inferential statistics) Survey weightings that were calibrated against population benchmarks (i.e., age, sex and area of usual residence) were used to account for the complex survey design [25,34]. Both base weight and 60 replicate weights have been incorporated into all estimations. Population characteristics and dietary intakes were reported as *n* (%), mean (standard error, SE), and median (interquartile range, IQR). Binary and ordinal logistic regressions were used to determine the likelihood of meeting dietary recommendations between the younger and older age groups, both in unadjusted and adjusted models. A directed acyclic graph was used to determine covariates in the adjusted analyses, which included BMI, country of birth, household type, level of education (postgraduate degree/diploma/certificate, graduate degree, certificate, or school qualification or lower), SEIFA, smoking status, alcohol (except when it was the outcome), physical activity and supplements use. Adjusted linear regression was performed to assess the mean difference between age groups for total and sub-component DGI score outcomes. Separate unadjusted and adjusted binary and ordinal logistic models were undertaken for the interaction between age categories (binary) and child in household (yes/no) to examine relationships with the meeting of dietary recommendations. Similar interactions were included in adjusted linear models with total and sub-component DGI score outcomes. All data were analysed using the statistical software SAS 9.4 (SAS Institute Inc., Cary, NC, USA).

## 3. Results

### 3.1. Participant Characteristics

Table 1 shows the demographic characteristics of the 2323 reproductive age women participating in the Australian Health Survey, Nutrition and Physical Activity, 2011–13. The mean (SE) age of the women was 33.9 (1.2) years and majority (71.1%) were born in Australia. Half of the women (49.1%) were aged 19 to 35 years with a mean (SE) age and BMI of 26.7 (0.8) years and 25.2 (0.8) kg/m^2^, respectively. The mean (SE) age and BMI of the older women (35 to 50 y) was 42.6 (0.5) years and 27.2 (0.7) kg/m^2^, respectively. A higher percentage of the older women tended to have overweight or obesity, and reported to live as a couple with children (Table 1).

### 3.2. Food Intake Compared to AGHE Recommendations among All Women and by Age Group

**Figure 1:**
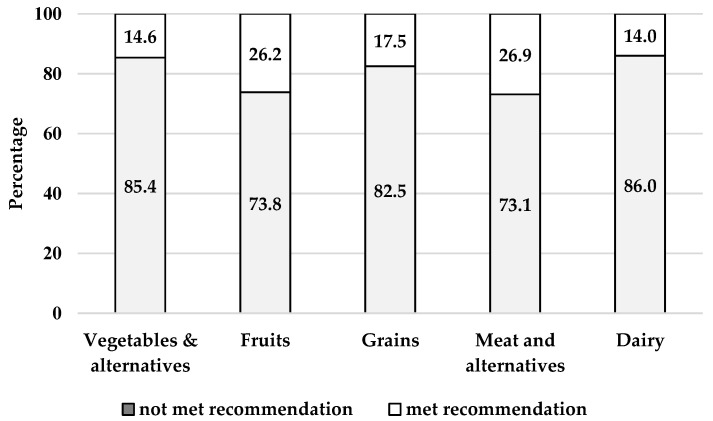
Adherence to food group recommendations in all reproductive age women (*n* = 2323). Australian Guide to Healthy Eating recommendations for vegetables & alternatives (≥5 servings), fruits (≥2 servings), grains (≥6 servings), meat and alternatives (≥2.5 servings), and dairy (≥2.5 servings).

Figure 1 displays the percentage of women meeting the AGHE recommended food group servings. Majority of women did not meet the minimum requirement for any of the five food groups. The highest percentage of women meeting recommendations was for meat and alternatives, and for fruit, at 26.9% and 26.2%, respectively. This was equivalent to a median (IQR) daily intake of meat and alternatives, and fruit, of 1.5 (0.7, 2.6) and 0.9 (0.0, 2.0) servings/day, respectively (Table 2). The lowest percentage of women who met recommendation was for vegetables (14.6%), equivalent to 2.1 (1.0, 3.7) servings/day (Table 2). Recommendations for grains and dairy was met by a respective 17.5% and 14.0%, of women. No significant differences were found between younger and older women in meeting AGHE recommendations (Table 3).

### 3.3. Food Intakes Compared to AMDR Recommendations in All Women and by Age Group

**Figure 2:**
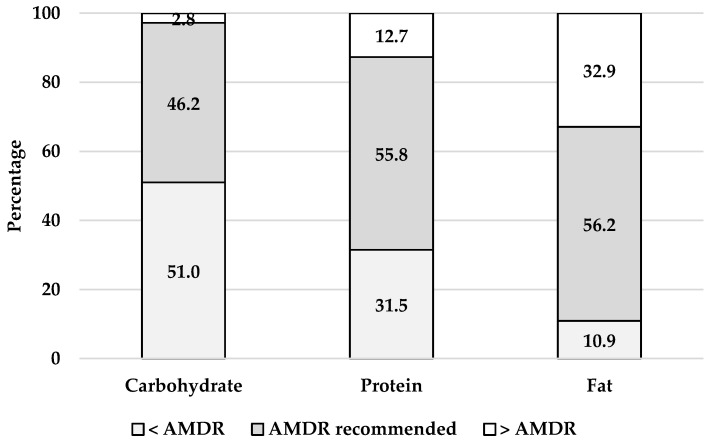
Percentage of daily energy from macronutrients in all reproductive age women (*n* = 2323). Acceptable macronutrient distribution range (AMDR) for carbohydrate (45–65%), protein (15–25%) and fat (20–35%).

The percentage of women meeting the AMDR is shown in Figure 2. Just over half of all women were within the AMDR for protein and fat and just under half were within the AMDR for carbohydrates. A third of women (31.5%) consumed less than the AMDR for protein but a third (32.9%) consumed higher than the AMDR for fat. There was no difference between younger and older women in meeting AMDR recommendations (Table 3).

### 3.4. Diet Quality and DGI Component Scores in All Women and by Age Group

Scores for both total DGI and its subcomponents are shown in Table 4. There was no significant difference for DGI total score between younger and older women. Compared to younger women, older women had higher DGI scores in limiting saturated fat, consuming low-fat milk, and limiting adding salt during cooking (Table 4).

### 3.5. Sub Group Analyses in Women with and without Children

For women in any age group, there was no difference in meeting the AGHE food group serving recommendations, AMDR guidelines, or DGI score, if women had children or not (Supplementary Tables S1 and S2).

## 4. Discussion

Using the largest and most-recent Australian National Nutrition and Physical Activity Survey, our results do not support our hypothesis that older women are more likely to meet dietary recommendations or have better diet quality than younger women of reproductive age. There was also no difference in meeting dietary recommendations whether women had children or not. Our study reiterates the overall inadequate diet quality of women in Australia, but extends previous studies showing that older women, or women who have children, have no superior diets compared to younger women or those without children.

Our findings are not unique to the Australian population of reproductive aged women. Studies in women from low income [35,36] and high income [37] countries have reported similar findings with low consumption of fruits and vegetables, and higher intakes of junk foods and discretionary choices. Compared to data collected from earlier Australian surveys, in 4349 women aged 18–46 years from the Australian Resilience for Eating and Activity Despite Inequality study, >90% failed to meet the recommended guidelines for vegetables, grains, lean meat and alternatives, and dairy foods [38]. Data from the Australian Longitudinal Study on Women’s Health (2001 to 2009) revealed that <2% of women aged 31–36 years or 50–55 years, met the Australian Dietary Guidelines recommendation of five daily servings of vegetables; and for women aged 31–36 years, less than one-third met recommendations for fruit and meat and alternatives [18]. The current analysis from the 2011 Australian Health Survey, reveals only 15% of women consumed adequate vegetable intake, with similar proportions of women still not meeting fruit, or meat and alternative groups compared to the Australian Longitudinal Study on Women’s Health. Thus, little progress has been made among reproductive age women meeting nutrition recommendations, and substantial changes to their dietary intake are needed to meet these.

Novel to our study is that we reveal consistency in the proportion of younger and older women meeting dietary guidelines, and no superior diet quality in older women, apart from scoring higher for some components of the DGI including limiting saturated fat, higher consumption of low fat milk, and lower added salt during cooking. Although limiting both saturated fat and added salt is recommended in the Australian Dietary Guidelines [30], and low fat milk is recommended to lower saturated fat intake [30], the extent to which these sub components contribute to overall diet quality cannot be established from the data. Nevertheless, the demonstration that older women are not consuming better quality diets is intriguing. There are clear links between advancing age and reproductive health. Physiologically, older women have diminished ovarian reserve [39] and shorter menstrual cycles [40], which impact fertility. More older women are entering pregnancy than what they were decades ago [41], frequently with higher body weight and a greater number of pre-existing conditions [42], which associate with poor reproductive health outcomes [42]. Older women also have higher rates of numerous risk factors for chronic diseases [24]. Given that many women are unaware of the importance of lifestyle choices when planning a pregnancy [43,44], and that there are a number of perceived barriers relating to dietary behaviours [45], action is required to increase women’s awareness and uptake of lifestyle advice and support [46].

Unique to our study is the report of no evidence of meeting dietary recommendations whether younger or older women had children or not. This has not been clearly assessed in previous studies. Parental influences play a large role in child feeding practices by deciding which foods are available and in what quantity [47,48]. Family eating habits have the greatest influence over young children’s diets [49], and one study showed that dietary indicators of mothers was a strong predictor of children’s dietary quality [50]. Although there is a large volume of research demonstrating the relationship between mothers’ and children’s food restraint and eating behaviours [51,52], no studies were found comparing dietary recommendations or quality between women with or without children. Our results reinforce the need for increased education to women and families to encourage healthy eating habits, as they are clear role models to their children. Our research also posits the need for research to examine relationships between mother-child diets.

Strengths of this study include the large nationally representative sample, generalisable to the broader Australian population of reproductive age women, and detailed sociodemographic and diet data. Specifically, the national survey collected food intake data in line with current Australian dietary recommendations, thereby providing easier translation of results. The systematic data collection methods employed within the Australian Health Survey allowed us to include appropriate confounding factors, reducing information bias. Limitations include the use of one-day dietary intake, thus not reflecting usual intake, along with a low sample of 24-hr recalls collected for Friday and Saturday [53]. This would likely underrepresent days where high intake of discretionary choices might be consumed. Although we adjusted analyses for several characteristics, the possibility of residual confounding impedes definitive conclusions about causality. The survey was conducted in 2011 and dietary intakes, along with changing societal behaviours such as prevalence of obesity and older maternal age, is likely to be different at present compared to 10 years ago.

## 5. Conclusions

In conclusion we report no differences between younger and older women of reproductive age in meeting dietary recommendations for food groups or macronutrients, and there was no difference in diet quality. Our findings reinforce the continued need for health promotion for women of reproductive age as a key priority to improve their own health but also that of future generations.

## Figures and Tables

**Table 1:** Characteristics of reproductive age women participating in the Australian Health Survey, Nutrition and Physical Activity, 2011–12.

	Characteristics	Frequency (%) or Mean (SE)		
		Total Population, *n* = 2323	19–35 Years, *n* = 1141	35–50 Years, *n* = 1182
Age (year)		33.9 (1.2) ^1^	26.7 (0.8) ^1^	42.6 (0.5) ^1^
		*n* = 1988	*n* = 1017	*n* = 971
BMI (kg/m^2^)		26.1 (0.4) ^1^	25.2 (0.8) ^1^	27.2 (0.7) ^1^
	Underweight	51 (3.1)	42 (4.6)	9 (1.3)
	Normal weight	946 (50.5)	522 (55.9)	424 (43.5)
	Overweight	508 (24.2)	231 (20.5)	277 (28.9)
	Obesity	483 (22.2)	222 (19.0)	261 (26.4)
	Australia	1706 (71.1)	860 (73.5)	846 (68.3)
Country of birth	Main English-speaking countries ^2^	224 (10.4)	93 (8.2)	131 (13.0)
	Other	393 (18.5)	188 (18.3)	205 (18.7)
	Person living alone	323 (7.2)	139 (6.1)	184 (8.6)
	Couple only	338 (13.6)	221 (17.4)	117 (9.0)
Household type	Couple family with children	965 (51.4)	397 (43.9)	568 (60.5)
	One parent family with children	445 (12.8)	199 (12.3)	246 (13.4)
	All other households ^3^	252 (14.9)	185 (20.3)	67 (8.5)
		*n* = 2300	*n* = 1132	*n* = 1168
	Postgraduate degree (Diploma/Certificate)	233 (9.2)	86 (7.3)	147 (11.4)
Level of education	Graduate degree	831 (36.5)	412 (36.1)	419 (36.9)
	Certificate	510 (24.0)	285 (27.8)	225 (19.4)
	School qualification or lower	726 (30.3)	349 (28.8)	377 (32.3)
	Quintile 1	418 (17.3)	233 (19.6)	185 (14.5)
	Quintile 2	420 (17.6)	218 (18.6)	202 (16.3)
SEIFA 2011—IRSD ^4^	Quintile 3	476 (21.6)	237 (23.0)	239 (19.9)
	Quintile 4	416 (18.6)	182 (16.0)	234 (21.8)
	Quintile 5	593 (24.9)	271 (22.8)	322 (27.4)
	Current smoker, daily	442 (16.5)	226 (18.1)	216 (14.6)
	Current smoker, weekly ^5^	41 (1.4)	25 (1.7)	16 (1.1)
Smoking status	Current smoker, less than weekly	18 (1.1)	15 (1.6)	3 (0.4)
	Ex-smoker	535 (22.4)	189 (16.2)	346 (29.8)
	Never smoked	1287 (58.6)	686 (62.4)	601 (54.1)
Meeting minimum physical activity requirement ^6^		*n* = 2312	*n* = 1136	*n* = 1176
	Yes	1236 (53.6)	626 (55.7)	610 (51.1)
	No	1076 (46.4)	510 (44.3)	566 (48.9)
Supplement use	Yes	714 (28.9)	295 (25.5)	419 (33.1)
	No	1609 (71.1)	846 (74.5)	763 (66.9)

^1^ Represents mean (SE); ^2^ Canada, Ireland, New Zealand, South Africa, United Kingdom and the United States of America; ^3^ All other households include Unrelated persons aged 15+ only and all other households, ^4^ Index of Relative Socio-Economic Disadvantage; ^5^ At least once a week but not daily; ^6^ Meeting the recommendation of physical activity for 150 min during the last week.

**Table 2:** Food group intakes for the whole population and by age group.

	Daily Servings, Median (IQR)			
		Total Population, *n* = 2323	19–35 Years, *n* = 1141	35–50 Years, *n* = 1182
	Vegetables & alternatives	2.1 (1.0, 3.7)	1.9 (1.0, 3.7)	2.1 (1.0, 3.8)
	Fruits	0.9 (0.0, 2.0)	0.9 (0.0, 2.0)	0.9 (0.0, 2.0)
Food groups (serving/d)	Grains	3.4 (2.1, 5.1)	3.4 (2.2, 5.2)	3.4 (1.9, 5.1)
	Meat & alternatives	1.5 (0.7, 2.6)	1.5 (0.6, 2.5)	1.6 (0.8, 2.7)
	Dairy	1.1 (0.4, 1.9)	1.1 (0.4, 1.9)	1.1 (0.5, 1.9)
	Carbohydrate	44.7 (37.2, 51.3)	45.3 (38.4, 52.1)	43.6 (35.0, 50.1)
Macronutrients percentage (%)	Protein	17.7 (13.9, 21.5)	17.0 (13.5, 21.1)	18.2 (14.5, 22.1)
	Fat	31.4 (25.5, 37.0)	31.5 (25.6, 37.4)	31.2 (25.4, 36.6)
	Percentage of energy from SFA	11.9 (8.7, 15.3)	12.2 (8.7, 15.3)	11.5 (8.5, 15.2)
Discretionary choices	Percentage of energy from free sugars ^1^	8.9 (4.7, 14.6)	9.9 (5.6, 15.8)	7.6 (4.0, 13.2)
	Sodium (mg/d)	1889.6 (1287.0, 2725.7)	1948.3 (1296.4, 2815.9)	1828.0 (1265.8, 2581.5)
	Alcohol (g/d)	0.0 (0.0, 0.1)	0.0 (0.0, 0.0)	0.0 (0.0, 13.6)
Dietary Guideline Index (total score)		76.3 (65.9, 85.8)	75.0 (64.4, 85.6)	77.5 (67.6, 86,1)

^1^ Free sugars include added sugars, sugar component of honey, fruit juice and fruit juice concentrates, based on the definition by the World Health Organization.

**Table 3:** Odds ratios for adherence to AGHE and AMDR recommendations ^1^.

	Reference	Unadjusted OR (95% CI)	Adjusted ^2^ OR (95% CI)
Vegetables & alternatives	<5 servings/day	1.00 (0.49, 2.04)	1.16 (0.28, 4.81)
Fruits	<2 servings/day	1.01 (0.63, 1.64)	1.16 (0.68, 1.96)
Grains	<6 servings/day	0.99 (0.33, 2.97)	1.02 (0.30, 3.44)
Meat & alternatives	<2.5 servings/day	0.88 (0.57, 1.35)	0.87 (0.51, 1.48)
Dairy	<2.5 servings/day	0.81 (0.43, 1.52)	0.70 (0.25, 1.98)
Alcohol	<40 g/day	1.92 (0.54, 6.71)	2.32 (0.45, 11.96)
Sugar	<10% daily energy intake ^3^	0.62 (0.25, 1.53)	0.69 (0.33, 1.44)
Sodium	<2000 mg/day	0.81 (0.48, 1.37)	0.82 (0.44, 1.52)
SFA	<10% daily energy intake	0.81 (0.48, 1.37)	0.82 (0.44, 1.52)
Carbohydrate	<45%	1.32 (0.80, 2.15)	1.13 (0.61, 2.08)
Protein	<15%	0.78 (0.50, 1.20)	0.75 (0.44, 1.28)
Fat	<20%	1.07 (0.70, 1.63)	1.07 (0.63, 1.82)

^1^ Reference was 35–50 years compared to 19–35 years; all statistical differences between groups in unadjusted and adjusted analyses were *p* > 0.05, ^2^ Adjusted for country of birth, household type, level of education, SEIFA, smoking status, alcohol (except when it was the outcome), BMI, physical activity and supplements use, ^3^ based on World Health Organization definition.

**Table 4:** Dietary Guideline Index (DGI) and its components, by age groups (*n* = 2323).

DGI	Mean (SE)		Mean Difference (SE) ^1,2^	*p*-Value
	19–35 Years (*n* = 1141)	35–50 Years (*n* = 1182)		
DGI (total score)	74.5 (2.5)	75.6 (1.7)	−0.54 (1.99)	0.79
DGI sub-components				
1. Food variety	1.9 (0.1)	2.3 (0.1)	−0.26 (0.27)	0.33
2. Vegetables	4.2 (0.2)	4.4 (0.3)	0.00 (0.49)	0.99
3. Fruit	4.8 (0.4)	4.8 (0.3)	0.12 (0.63)	0.84
4. Cereal (total)	3.6 (0.4)	3.3 (0.2)	0.15 (0.63)	0.80
4a. Serves per day	2.3 (0.2)	2.1 (0.2)	0.17 (0.37)	0.65
4b. Mostly wholegrain	1.2 (0.2)	1.2 (0.2)	−0.01 (0.31)	0.97
5. Meat and Alternatives (total)	7.0 (0.2)	7.3 (0.1)	−0.24 (0.33)	0.47
5a. Serves per day	2.5 (0.1)	2.8 (0.1)	−0.20 (0.20)	0.31
5b. Mostly lean	4.5 (0.1)	4.5 (0.1)	−0.03 (0.25)	0.89
6. Dairy and alternatives	4.9 (0.4)	5.0 (0.2)	−0.17 (0.41)	0.67
7. Fluid intake (total)	8.4 (0.1)	8.6 (0.2)	−0.17 (0.23)	0.44
7a. Serves per day	3.9 (0.1)	4.2 (0.2)	−0.24 (0.21)	0.26
7b. Mostly water	4.5 (0.1)	4.5 (0.1)	0.06 (0.11)	0.54
8. Limit discretionary foods	3.4 (0.7)	3.8 (0.3)	−0.55 (0.73)	0.45
9. Limit saturated fat (total)	7.8 (0.3)	8.5 (0.2)	−0.69 (0.33)	0.04
9a. Mostly trimmed meat	4.4 (0.1)	4.4 (0.1)	−0.07 (0.21)	0.74
9b. Mostly low-fat milk	3.4 (0.3)	4.0 (0.1)	−0.62 (0.28)	0.03
10. Moderate unsaturated-fat	7.9 (0.4)	7.6 (0.5)	0.36 (1.05)	0.73
11. Limit added salt (total)	5.9 (0.2)	6.1 (0.4)	−0.28 (0.32)	0.38
11a. During cooking	2.2 (0.2)	2.7 (0.3)	−0.47 (0.21)	0.03
11b. Added at the table	3.6 (0.1)	3.4 (0.2)	0.19 (0.22)	0.40
12. Limit extra sugar	6.2 (0.5)	6.8 (0.3)	−0.50 (0.56)	0.37
13. Limit alcohol	9.4 (0.3)	8.9 (0.2)	0.57 (0.45)	0.58

^1^ Reference was 35–50 years, ^2^ Adjusted for country of birth, household type, level of education, SEIFA, smoking status, alcohol (except for when it was the outcome), BMI, physical activity and supplements use.

## Data Availability

Microdata products are available to approved users. Data are available at https://www.abs.gov.au/websitedbs/D3310114.nsf/home/MicrodataDownload, and upon request to: microdata.access@abs.gov.au.

## Supplementary Materials

The following are available online at https://www.mdpi.com/article/10.3390/nu13113830/s1, Supplementary Table S1: Likelihood for adherence to AGHE and AMDR recommendations among women without compared to with child. Supplementary Table S2: Dietary Guideline Index (DGI) among women without compared to with child
