# Supplementary material for: Do Older Women of Reproductive Age Have Better Diet Quality than Younger Women of Reproductive Age?
Source: Nutrients. 2021 Oct 27;13(11):3830. doi: 10.3390/nu13113830 (PMC8625416; doi:10.3390/nu13113830)
Supplement: Supplementary file 1 [file nutrients-13-03830-s001.zip › nutrients-1374042-supplementary.pdf]

Supplementary Table S1. Likelihood for adherence to AGHE and AMDR recommendations among women without compared to with child.

		19-35 y		35-50 y	
	Reference	Unadjusted OR (95% CI)	Adjusted[1] OR (95% CI)	Unadjusted OR (95% CI)	Adjusted[1] OR (95% CI)
Vegetables & alternatives	< 5 servings/day	1.06 (0.49, 2.28)	0.89 (0.32, 2.41)	1.21 (0.25, 5.73)	1.04 (0.10, 10.44)
Fruits	< 2 servings/day	1.17 (0.56, 2.46)	1.01 (0.43, 2.38)	1.06 (0.54, 2.07)	0.89 (0.33, 2.41)
Grains	< 6 servings/day	1.18 (0.47, 2.92)	1.15 (0.33, 3.93)	1.15 (0.17, 7.78)	1.18 (0.13, 10.37)
Meat & alternatives	< 2.5 servings/day	0.80 (0.38, 1.64)	0.69 (0.32, 1.49)	1.01 (0.27, 3.73)	0.80 (0.30, 2.13)
Dairy	< 2.5 servings/day	1.17 (0.26, 5.13)	1.19 (0.18, 7.92)	0.81 (0.44, 1.51)	0.83 (0.35, 1.97)
Alcohol	< 40 g/day	0.39 (0.10, 1.42)	0.41 (0.10, 1.54)	0.72 (0.17, 3.04)	0.55 (0.13, 2.30)
Sugar	< 10% daily energy intake	0.92 (0.58, 1.48)	0.83 (0.44, 1.56)	0.90 (0.47, 1.73)	0.90 (0.43, 1.90)
Sodium	< 2000 mg/day	1.08 (0.54, 2.19)	0.96 (0.45, 2.03)	0.97 (0.39, 2.38)	0.97 (0.45, 2.07)
SFA	< 10% daily energy intake	1.14 (0.54, 2.37)	0.92 (0.44, 1.88)	1.09 (0.38, 3.13)	0.95 (0.41, 2.21)
Carbohydrate	< 45%	0.87 (0.45, 1.67)	0.91 (0.50, 1.66)	1.06 (0.30, 3.66)	1.16 (0.41, 3.27)
Protein	< 15%	0.90 (0.49, 1.66)	1.06 (0.59, 1.88)	0.90 (0.24, 3.29)	0.98 (0.24, 4.04)
Fat	< 20%	0.66 (0.31, 1.40)	0.74 (0.35, 1.54)	1.03 (0.58, 1.82)	1.16 (0.39, 3.39)

[1]Adjusted for country of birth, household type, level of education, SEIFA, smoking status, alcohol (except for outcome: Alcohol), BMI, physical activity and number of supplements

Supplementary Table S2. Dietary Guideline Index (DGI) among women without compared to with child.

DGI	19-35 y				35-50 y			
	Mean (SE)		Mean difference (SE)	P-value	Mean (SE)		Mean difference (SE)	P-value
	No child (n=545)	Have child (n=596)			No child (n=368)	Have child (n=814)		
DGI (total score)	76.0 (3.1)	74.6 (2.7)	1.44 (2.79)	0.607	73.1 (3.8)	77.0 (3.1)	-3.89 (2.51)	0.13
DGI sub-components								
1. Food variety	2.0 (0.2)	2.0 (0.2)	-0.01 (0.20)	0.942	2.2 (0.5)	2.4 (0.3)	−0.20 (0.33)	0.63
2. Vegetables	4.6 (0.6)	4.9 (0.7)	-0.30 (0.52)	0.562	4.8 (1.2)	4.8 (0.7)	−0.05 (0.77)	0.95
3. Fruit	5.0 (0.8)	4.9 (0.8)	0.10 (0.69)	0.879	4.4 (1.4)	5.1 (0.8)	−0.66 (1.50)	0.66
4. Cereal (total)	3.8 (0.6)	3.7 (0.6)	0.07 (0.47)	0.883	3.5 (1.0)	3.7 (0.7)	−0.11 (0.55)	0.84
4a. Serves per day	2.4 (0.3)	2.5 (0.3)	-0.08 (0.30)	0.801	2.3 (0.9)	2.3 (0.4)	−0.040 (0.60)	0.95
4b. Mostly wholegrain	1.4 (0.4)	1.2 (0.4)	0.15 (0.34)	0.669	1.3 (0.5)	1.3 (0.4)	−0.07 (0.40)	0.85
5. Meat and Alternatives (total)	6.7 (0.8)	6.7 (0.6)	0.00 (0.80)	0.991	6.9 (0.8)	7.0 (0.6)	−0.11 (0.50)	0.82
5a. Serves per day	2.4 (0.4)	2.5 (0.4)	-0.12 (0.39)	0.761	2.6 (0.5)	2.7 (0.4)	−0.16 (0.41)	0.70
5b. Mostly lean	4.3 (0.5)	4.2 (0.3)	0.13 (0.44)	0.775	4.3 (0.4)	4.3 (0.3)	0.05 (0.16)	0.76
6. Dairy and alternatives	5.2 (0.8)	4.7 (0.6)	0.49 (0.85)	0.564	4.8 (0.9)	5.2 (0.6)	−0.36 (1.07)	0.74
7. Fluid intake (total)	8.9 (0.3)	8.6 (0.3)	0.35 (0.26)	0.192	8.6 (0.4)	9.0 (0.4)	−0.33 (0.36)	0.37
7a. Serves per day	4.3 (0.3)	4.0 (0.2)	0.26 (0.18)	0.160	4.2 (0.4)	4.4 (0.3)	−0.13 (0.26)	0.62
7b. Mostly water	4.7 (0.2)	4.6 (0.2)	0.09 (0.20)	0.654	4.4 (0.4)	4.6 (0.2)	−0.20 (0.39)	0.61

8. Limit discretionary foods	3.1 (1.1)	3.0 (0.9)	0.061 (0.75)	0.935	3.5 (0.9)	3.7 (1.3)	−0.16 (1.23)	0.90
9. Limit saturated fat (total)	7.9 (0.6)	7.3 (0.6)	0.63 (0.41)	0.130	7.9 (0.9)	8.3 (0.5)	−0.40 (0.94)	0.67
9a. Mostly trimmed meat	4.4 (0.4)	4.1 (0.4)	0.27 (0.25)	0.270	4.2 (0.4)	4.4 (0.4)	−0.18 (0.38)	0.64
9b. Mostly low-fat milk	3.5 (0.4)	3.2 (0.4)	0.36 (0.39)	0.358	3.7 (0.7)	4.0 (0.4)	−0.22 (0.67)	0.75
10. Moderate unsaturated-fat	8.5 (0.9)	8.1 (0.8)	0.34 (0.77)	0.662	7.9 (1.3)	7.8 (1.1)	0.14 (0.83)	0.87
11. Limit added salt (total)	5.1 (0.6)	5.1 (0.4)	-0.01 (0.47)	0.975	5.9 (0.7)	5.3 (0.6)	0.62 (0.45)	0.17
11a. During cooking	1.4 (0.3)	1.5 (0.3)	-0.12 (0.28)	0.666	2.2 (0.3)	1.9 (0.4)	0.37 (0.35	0.29
11b. Added at the table	3.7 (0.5)	3.6 (0.3)	0.11 (0.35)	0.759	3.7 (0.5)	3.4 (0.4)	0.26 (0.27)	0.36
12. Limit extra sugar	6.0 (1.2)	6.4 (1.2)	−0.41 (0.72)	0.568	7.2 (1.3)	6.6 (1.4)	0.58 (2.18)	0.79
13. Limit alcohol	9.1 (0.4)	9.6 (0.4)	−0.55 (0.30)	0.073	8.5 (0.9)	9.1 (0.5)	−0.54 (0.73)	0.46

All analyses adjusted for country of birth, household type, level of education, SEIFA, smoking status, alcohol (except for the outcome: Limit alcohol), BMI, physical activity and number of supplements
